# The influence of specific groups of osteogenic supplements on the differentiation of MC3T3-E1 cells over time

**DOI:** 10.1007/s11626-026-01194-8

**Published:** 2026-07-20

**Authors:** Erik Betzold, Monika Bubel, Norbert Pütz, Matthias Hannig, Emmanouil Liodakis, Tim Pohlemann, Wolfgang Metzger

**Affiliations:** 1 Department of Trauma, Hand and Reconstructive Surgery, Saarland University, Building 57, 66421 Homburg, Germany; 2 Clinic of Operative Dentistry, Periodontology and Preventive Dentistry, Saarland University, Building 73, 66421 Homburg, Germany

**Keywords:** Bone, MC3T3-E1, Osteogenic differentiation, Osteogenic supplements

## Abstract

The setup of experimental designs in basic bone research begins with selecting appropriate osteogenic supplements to induce differentiation. Many studies describe the use of the supplements ascorbic acid, dexamethasone, and β-glycerophosphate in varying combinations and concentrations. To clarify their individual and combined effects on MC3T3-E1 cells, we conducted a detailed study focusing on cell morphology, mineralization, and gene expression of key osteogenic markers at four time points. Cell morphology was analyzed using light and scanning electron microscopy. Mineralization was assessed via Alizarin Red staining for calcium and von Kossa staining for phosphate. Additionally, energy-dispersive X-ray analysis was used to detect calcium and phosphorus in the cell layer. Sirius Red staining was applied to characterize the fiber formation observed under electron microscopy. To evaluate the influence of the supplements on gene expression, real-time quantitative PCR was performed for the osteogenic markers alkaline phosphatase, bone sialoprotein, bone morphogenetic protein 2, collagen 1a2, osteocalcin, osterix, and runt-related transcription factor 2. Osteogenic differentiation of MC3T3-E1 cells could be induced by application of ascorbic acid and β-glycerophosphate leading to upregulation of osteogenic markers, changes in cellular morphology, and mineralizing activity. Nevertheless, this effect could be further increased by additional use of dexamethasone. While most osteogenic marker genes were already upregulated on day 7, we did not see the first signs of mineralization until day 14. We recommend using ascorbic acid, β-glycerophosphate, and dexamethasone in order to get an osteogenic phenotype of MC3T3-E1 on day 14 at the earliest.

## Introduction

Due to our aging population, the incidence of bone diseases such as osteoporosis and fragility fractures is expected to increase in the near future. According to Kanis *et al*., fragility fractures in the EU will increase from 3.5 million in 2010 to 4.5 million in 2025 (Kanis *et al*. 2019). These rapidly increasing numbers highlight the need for a better understanding of bone metabolism physiology, which will help to generate new therapeutic options for patients. In the history of in vitro bone cell research, cells are typically cultured in a two-dimensional setup and supplemented with osteogenic agents such as ascorbic acid (AA), β-glycerophosphate (β-GP), dexamethasone (DX), vitamin K, or bone morphogenetic protein 2 (BMP2). These supplements support the differentiation of cells such as mesenchymal stem cells or osteoprogenitor cells into mature osteoblasts (Tenenbaum 1981; Hong *et al*. 2009; Brauer *et al*. 2016; Wang *et al*. 2020). In this study, we investigated the influence of three osteogenic supplements (AA, DX, and β-GP) on the osteogenic differentiation of MC3T3-E1 subclone 4 over time. The MC3T3-E1 murine preosteoblastic cell line, which is derived from the calvaria of nine newborn C57BL/6 mice (Kodama *et al*. 1981) is an established in vitro model for studying osteogenic differentiation (Sudo *et al*. 1983; Czekanska *et al*. 2012). The cells’ high proliferative capacity and homogeneous character allow comparative studies, and of course, the possibility to study osteogenic differentiation in vitro. However, it is clear that interspecies differences must be taken into account (Czekanska *et al*. 2012).

We examined the supplements both alone and in all possible combinations to gain a structured overview of the three main supplements, which are often part of the multitude of heterogeneous osteogenic differentiation protocols described in the literature. AA is known to be an essential cofactor in the hydroxylation process of procollagen. This step is necessary to generate functional collagen, which has been shown to be a prerequisite for osteogenic differentiation (Franceschi and Iyer 1992). In 1996, Takeuchi *et al*. demonstrated that matrix-associated collagen binds to the α2β1 integrin receptor, thereby supporting osteogenic differentiation in MC3T3-E1 cells (Takeuchi *et al*. 1996). In addition to the well-known mechanism by which AA influences osteogenic differentiation via collagen, newer studies have reported other ways in which AA influences bone metabolism. In 2022, Thaler *et al*. reported that AA epigenetically influences cells in the process of osteogenic differentiation. AA accomplishes this by demethylating the histones H3k9me3 and H3k27me3, which opens specific chromatin areas that stimulate the expression of osteogenic genes. Another pro-osteogenic influence of AA is the activation of ten-eleven translocation (TET) enzymes, which catalyze the hydroxylation of 5-methylcytosine to 5-hydroxymethylcytosine. The accumulation of 5-hydroxymethylcytosine promotes chromatin opening and gene expression, thereby supporting osteogenic differentiation (Thaler *et al*. 2022). The bone is made up of many inorganic substances, such as calcium and phosphate (Pilitsis *et al*. 2002). During in vitro osteogenic differentiation, alkaline phosphatase (ALPL) cleaves β-GP, which then serves as a source of phosphate. Phosphate is one of the main inorganic components of bone (Makris *et al*. 2023; Garimella *et al*. 2006). The mechanism by which DX affects osteogenic differentiation is extremely broad, as it influences cells through various pathways (Langenbach and Handschel 2013). Despite the growing body of knowledge on osteogenic differentiation, many aspects of this complex process remain unknown. Thus, we cultured the MC3T3-E1 subclone 4 in a two-dimensional setup and analyzed the influence of the supplements, both individually and in combination, on the cells’ morphology and genetics at days 7, 14, 21, and 28. We analyzed the morphology of the cells using light and scanning electron microscopy. To evaluate the cells’ mineralizing activity, we stained them with Alizarin Red (AR) to identify the deposition of calcium both qualitatively and quantitatively. In addition, we used von Kossa (VK) staining for the qualitative detection of phosphate. Furthermore, the deposition of calcium and phosphorus was measured by energy dispersive X-ray (EDX) analysis. Finally, we quantified the expression of the osteogenic marker genes alkaline phosphatase (*ALPL*), bone sialoprotein (*IbSp*), bone morphogenetic protein 2 (*BMP2*), collagen type I alpha 2 (*COL1A2*), osteocalcin (*BGLAP*), osterix (*SP7*), and Runt-related transcription factor 2 (*RUNX2*) by real-time quantitative polymerase chain reaction (qPCR).

## Material and methods

### Cell culture

MC3T3-E1 (subclone 4) cryocultured cells were purchased from the American Type Culture Collection (ATCC®, Manassas, VA, Cat. No. CRL-2593, Lot No. 70019054) at passage 17. These cells were then expanded avoiding confluence and stored in liquid nitrogen as a master cell bank to ensure an adequate supply of cryovials for the entire study. Specifically, 1.4 × 10^6^ cells were seeded with a density of 80 cells/mm^2^. After 96 h, 6.03 × 10^6^ cells were harvested and seeded again at a density of 49 cells/mm^2^. After a further 96-hour incubation period, we obtained 40.8 × 10^6^ cells, which were stored in 81 cryovials with approximately 500,000 cells each as our master cell bank.

During the course of the study, the cells were cultivated in T75 cell culture flasks with alpha minimum essential medium (α-MEM) without AA (Pan-Biotech, Aidenbach, Germany, Cat. No. P04-21505), supplemented with 10% fetal calf serum (Pan-Biotech, Cat. No. P30-3306) and 1% of penicillin/streptomycin (Sigma-Aldrich, St. Louis, MO), until sub-confluency was reached. Standard trypsinization protocols were used to detach the cells, and cell counting was carried out manually with a hemocytometer. For this study, only cells up to passage 25 were used.

### Induction of osteogenic differentiation

Because the incubation time was up to 28 day, the cells were seeded in 24- or six-well plates (Greiner Bio-One, Frickenhausen, Germany), which were coated with collagen to prevent cell detachment. We added 190 µl or 960 µl of a sterile collagen solution (derived from rat tail, *c* = 100 µg/ml in 0.1% acetic acid, Corning Costar, Corning, NY) to each well. The wells were then incubated for 2 h under cell culture conditions. Then, the solution was removed, and the plates were allowed to dry under sterile conditions in the laminar flow hood overnight. The plates were stored in the refrigerator at 4 °C until use. Before seeding the cells (200 cells/mm^2^), we rinsed the wells with sterile phosphate-buffered saline (PBS, Oxoid Limited, Basingstoke, UK).

The cells were seeded on day −1 in an unsupplemented cell culture medium and reached approximately 100% confluence the next day. On day 0, osteogenic differentiation was induced by switching to an osteogenic induction medium containing the supplements AA (Fisher Scientific, Loughborough, UK), DX (Sigma Aldrich), and β-GP (Calbiochem, Darmstadt, Germany). The cell culture medium was mixed with the osteogenic supplements for all variations (Table 1). Unsupplemented cell culture medium served as the negative control (NC). The medium was changed three times a week, and cell morphology was checked and documented throughout the induction process using a Nikon Eclipse TS100 phase contrast microscope (objective ×10/0.25, Ph1 ADL; Nikon Corporation, Tokyo, Japan) and a Sony Cybershot 3.0 (Sony Corporation, Tokyo, Japan).

**Table 1.** Composition of the different supplement cocktails for each group

Group	Supplements
NC	-
AA	Ascorbic acid 50 µg/ml
DX	Dexamethasone 10 nM
β-GP	β-Glycerophosphate 10 mM
AA + DX	Ascorbic acid 50 µg/ml Dexamethasone 10 nM
AA + β-GP	Ascorbic acid 50 µg/ml β-Glycerophosphate 10 mM
DX + β-GP	Dexamethasone 10 nM β-Glycerophosphate10 mM
AA + DX + β-GP	Ascorbic acid 50 µg/ml Dexamethasone 10 nM β-Glycerophosphate 10 mM

### von Kossa staining

VK staining is used to detect phosphate and involves a two-step reaction. First, silver cations react with the deposited phosphate, which are then reduced to black metallic silver (Schneider 2021). The cell culture medium was removed, and the cells were rinsed with PBS before fixation. Then, an ice-cold mixture of 95% ethanol (BCD Chemie GmbH, Hamburg, Germany) and 5% isopropanol (Fisher Scientific) was added to the cell layer and left for 60 min at 4°C. After removing the fixative, the cells were rinsed with distilled water. The following steps were performed in the dark because the chemical reaction is light-sensitive. A freshly prepared silver nitrate solution (*c* = 2.5% in distilled water; Merck, Darmstadt, Germany) was added to the cells, which were then incubated for 20 min. Then, the silver nitrate solution was removed, and the cells were washed with distilled water again. The silver ions that bound to the phosphate in the mineralized areas were reduced by adding a hydroquinone solution (*c* = 5% in distilled water) for 2 min. Finally, a 5% sodium thiosulfate solution (Merck) in distilled water was added to stop the reaction. The groups were then analyzed macroscopically and microscopically for black-stained areas indicating mineralization.

### Alizarin Red staining

AR staining is an additional staining to detect mineralization. AR forms a chelate complex with calcium, which results in a red AR-calcium complex (Bernar *et al*. 2022). To prepare the staining solution, 2 g of Alizarin Red (Abcam, Cambridge, UK) were mixed with 80 ml of distilled water for 60 min using a rotary mixer. The solution was then filtered, and the pH was adjusted to 4.1 and 4.3. The solution was prepared the day before staining and stored overnight at 4 °C, covered with aluminum foil. To fix the cells, the medium was removed, and the cells were rinsed with PBS. Then, the cells were fixed with 4% paraformaldehyde (PFA, Carl Roth GmbH, Karlsruhe, Germany) in PBS for 15 min. After fixation, the cells were rinsed with distilled water and incubated for 30 min under agitation with the AR staining solution in the dark. The staining solution was then removed, and the cells were carefully rinsed twice with distilled water. The groups were then analyzed macroscopically and microscopically for the presence of red-stained areas, which indicate the presence of calcium.

### Alizarin Red quantification

Unlike VK staining, AR staining allows for elution and photometric quantification. The cells were therefore cultured in six-well plates and stained as described above (*n* = 5). After removing the unattached stain, the bound AR stain was eluted by adding 800 µl of acetic acid (10%) to each well and incubating under agitation for 30 min. The cells were then scraped off with a rubber policeman (NeoLab Migge Laborbedarf-Vertriebs GmbH, Heidelberg, Germany). The solution, along with the detached cells, was transferred to a 1.5-ml reaction tube and mixed thoroughly for 30 s. The reaction tube was incubated at 85 °C for 10 min and then cooled on ice for 5 min afterwards. To sediment cellular debris, the reaction tube was centrifuged for 20 min at 15,000*g* (Minifuge, Eppendorf, Hamburg, Germany). Then, a volume of 500 µl of the supernatant was transferred to a fresh reaction tube and mixed with 200 µl of ammonium hydroxide to adjust the pH to 4.1–4.5. Finally, 150 µl of the pH-adjusted solution was transferred into the cavities of a 96-well plate in duplicate for each group. Absorbance was measured at a wavelength of 405 nm with five flashes and a reference wavelength of 690 nm using the microplate reader Infinite M200 Pro (Tecan Group, Männedorf, Switzerland). Samples with a measured relative absorbance higher than 2.5 were diluted with a mixture of 500 µl of 10% acetic acid and 200 µl of ammonium hydroxide.

### Scanning electron microscopy and energy-dispersive X-ray analysis

In addition to the light microscopy analysis performed on days 7, 14, 21, and 28, the morphology of the cells was examined in detail using scanning electron microscopy (SEM) at the same time points. EDX analysis was performed on days 14, 21, and 28 to quantify the elemental composition. MC3T3-E1 cells were cultivated at a density of 200 cells/mm^2^ on sterilized coverslips (20 × 20 mm; Paul Marienfeld GmbH & Co. KG, Lauda-Königshofen, Germany; Cat. No. No. 106042) in six-well plates until the day of analysis. After incubating the cells, the medium was removed, and the cells were rinsed with PBS. The cells were fixed with 2% glutaraldehyde (Electron Microscopy Sciences, Hatfield, PA) in 0.1 M cacodylate buffer (Carl Roth GmbH, pH 7.4) for 10 min with agitation, followed by washing the cells three times in 0.1 M cacodylate buffer. For dehydration, the cells were incubated for 10 min in each concentration of an ascending ethanol series (3 × 70%, 1 × 80%, 1 × 90%, 1 × 96%, 1 × 100%). Dehydration was completed with an incubation in hexamethyldisilazane (HMDS, Carl Roth GmbH). First, a 1:1 mixture of HMDS and 100% ethanol (water-free) was pipetted onto the cells for 10 min, followed by an incubation in pure HMDS for 10 min. This last step was performed twice, after which the cells were stored under the hood overnight to allow for complete evaporation of the HMDS. Then, the coverslips with the dehydrated cell layers were transferred onto regular SEM pin stubs (Micro to Nano, Haarlem, Netherlands) with conductive adhesive tape and sputtered with carbon (Balzers Union, Balzers Liechtenstein). Samples intended for EDX analysis were sputtered only with carbon. Samples intended for cellular morphology analysis were additionally sputtered with gold (Bal-Tec GmbH, Schalksmühle, Germany). The samples were measured with a JEOL JSM-IT210 (JEOL GmbH, Freising, Germany) in backscattered electron mode with an acceleration voltage of 10 kV for EDX analysis. Five randomly selected areas were measured for each sample, with no preference given to morphologically conspicuous areas. Representative areas of the cell layers morphology were documented by SEM analysis with a FEI XL 30 ESEM FEG (FEI Company, Hillsboro, OR).

### Sirius Red staining

The deposition of collagen by cells during osteogenic differentiation can easily be monitored using Sirius Red (SR) staining. To identify and quantify the amount of collagen produced by the cells, an SR staining was performed in six-well plates on day 7 for all groups. The medium was removed, and the cells were rinsed with PBS. Then, the cells were fixed with 4% PFA in PBS and incubated under agitation for 10 min. The cell layer was then incubated in a dark environment at room temperature for 60 min under agitation in a staining solution consisting of 2 ml of SR (0.1%, Sigma Aldrich) dye dissolved in saturated 1.3% picric acid (Sigma Aldrich). After removing the staining solution, unbound dye was removed by washing the cell layer with 0.01 M hydrochloric acid four to five times. The staining solution attached to the collagen fibers was eluted by incubating the cells with 2 ml of 0.1 M sodium hydroxide for 30 min under agitation. For final quantification, 200 µl of the eluted solution was transferred into 96-well plates in duplicate and measured at 540 nm with five flashes and a reference wavelength of 690 nm in a microplate reader (Tecan Group). Samples with a relative absorbance higher than 2.5 were diluted accordingly with 0.1 M sodium hydroxide.

### Gene expression analysis

MC3T3-E1 cells were differentiated according to the protocol until days 7, 14, 21, and 28 to quantify the expression of typical osteogenic marker genes. The cells were harvested by scrubbing them with rubber policemen, after which the cell suspension was transferred into a 15-ml Falcon tube. The plate was rinsed with 3 ml of PBS which was then transferred into the 15-ml Falcon tube as well. After centrifugation at 300*g* for 4 min, the supernatant was removed, and the cells were incubated twice with 300 µl of RLT buffer (Qiagen, Hilden, Germany). Then, the cells were transferred into Precelly® tubes (Sarstedt AG & Co. KG, Nümbrecht, Germany), which were filled with 0.5 g of glass beads (Sigma-Aldrich, diameter 425–600 µm). The cells were completely lysed using a FastPrep-24 instrument (MP Biomedicals, Santa Ana, CA) at a speed of 6.5 m/s for 45 s. After lysis, the cells were centrifuged at 12,000*g* for 2 min in a Minifuge (Eppendorf AG, Hamburg, Germany). RNA was isolated from the supernatant using the RNeasy Mini Kit (Qiagen) according to the manufacturer’s instructions. The RNA concentration and purity were quantified using a NanoDrop One (Thermo Fisher Scientific, Waltham, MA). Transcription of RNA to cDNA was performed using the High Capacity cDNA Reverse Transcription Kit (Thermo Fisher Scientific) with an absolute amount of 500 ng of RNA per reaction. Three samples were prepared for each supplement group: two with reverse transcriptase and one without, serving as an internal negative control. qPCR was then performed on a StepOnePlus Real-Time PCR System (Applied Biosystems, Waltham, MA). The following TaqMan™ assays (Thermo Fisher Scientific), listed in Table 2, were used along with the associated TaqMan™ Gene Expression Master Mix (Thermo Fisher Scientific): To standardize, we used hypoxanthine phosphoribosyltransferase 1 (HPRT1), hydroxymethylbilane synthase (HMBS), and succinate dehydrogenase complex subunit A (SDHA) as housekeeping genes. We normalized each group to its housekeeping genes using the ΔCT method. We performed relative quantification of gene expression using the ΔΔCT method (Schmittgen and Livak 2008). To illustrate the changes in gene expression for each gene compared to its respective NC, we calculated the RQ value using the formula RQ = 2^(-ΔΔCT)^. RQ values < 1 illustrate a downregulated gene expression whereas RQ values > 1 demonstrate upregulated gene expression. Due to mathematical laws, it is not possible to calculate the standard deviation directly from the RQ value. Therefore, we calculated an RQ range via the formula proposed by Livak and Schmittgen. 2^−(mean^ ^ΔΔCT ± standard deviation ΔΔCT)^ as a measure of error (Livak and Schmittgen 2001).

**Table 2.** List of TaqMan™ assays used in the qPCR

Gene	Gene abbreviation	Assay ID	Amplicon length (bp)
Collagen 1a2	*Col1a2*	Mm00483888_m1	67
Bone sialoprotein	*IbSp*	Mm00492555_m1	98
Osteocalcin	*BGLAP*	Mm03413826_mH	110
Runt-related transcription factor 2	*RUNX2*	Mm00501584_m1	91
Osterix	*SP7*	Mm00504574_m1	137
Alkaline phosphatase	*ALPL*	Mm00475834_m1	65
Bone morphogenetic protein 2	*BMP2*	Mm01340178_m1	58
Hydroxymethylbilane synthase	*HMBS* Housekeeping gene	Mm01143545_m1	81
Hypoxanthine phosphoribosyltransferase 1	*HPRT1* Housekeeping gene	Mm03024075_m1	131
Succinate dehydrogenase complex subunit A	*SDHA* Housekeeping gene	Mm01352366_m1	82

### Statistical analysis

All quantitative results are shown as means with standard deviation unless otherwise noted. We used SigmaStat Version 13.0 (Systat Software, Inc., San Jose, CA) to perform one-way ANOVA tests followed by a Student–Newman–Keuls post hoc test to detect significant differences (*p* < 0.05). If the tests for equality of variance or normal distribution alone or both failed, a Kruskal–Wallis test with a Student–Newman–Keuls post hoc test was performed. When testing two groups against each other, an unpaired *t*-test was performed. For evaluating the qPCR, a Dunnett post hoc test was performed after a one-way ANOVA or Kruskal–Wallis based on the ΔCT values. GraphPad Prism version 10.2.3 (GraphPad Software, San Diego, CA) was used to illustrate the results, and a compact letter display was used to show significant differences (*p* < 0.05) between groups. The compact letter display allocates one or more small letters to a column. If two compared groups shared at least one small letter, there was no significant difference between them. If two groups did not share any small letter, they differed significantly from one another.

## Results

### Cell morphological changes of MC3T3-E1 cells during osteogenic differentiation

**Figure. 1:**
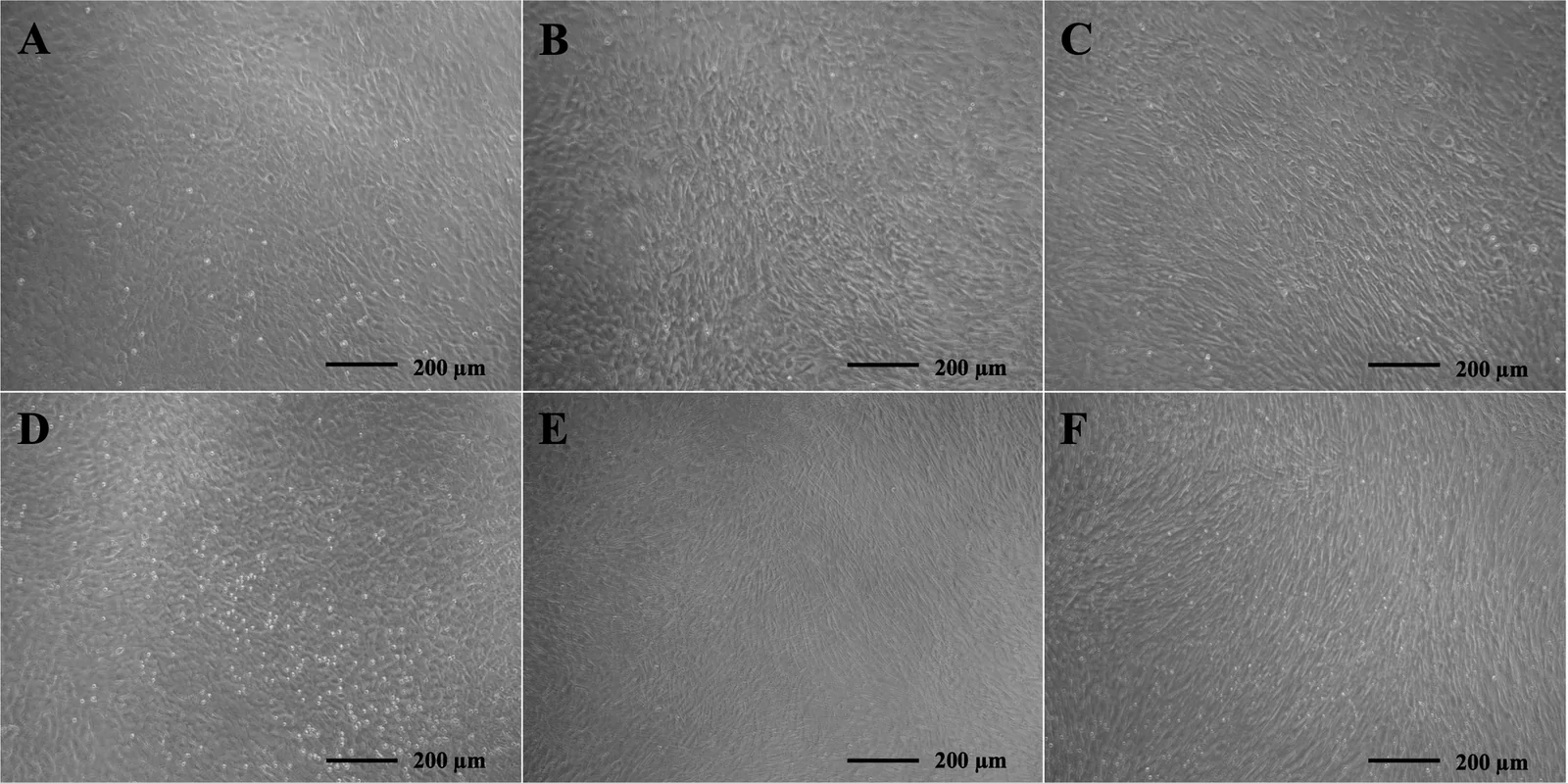
Microscopic images of MC3T3-E1 cells on day 3 (*A*–*C*) and day 6 (*D*–*F*) supplemented with different cocktails. NC without supplements (*A* + *D*). Cells supplemented with AA (*B* + *E*). Cells supplemented with AA + DX + β-GP (*C* + *F*).

**Figure. 2:**
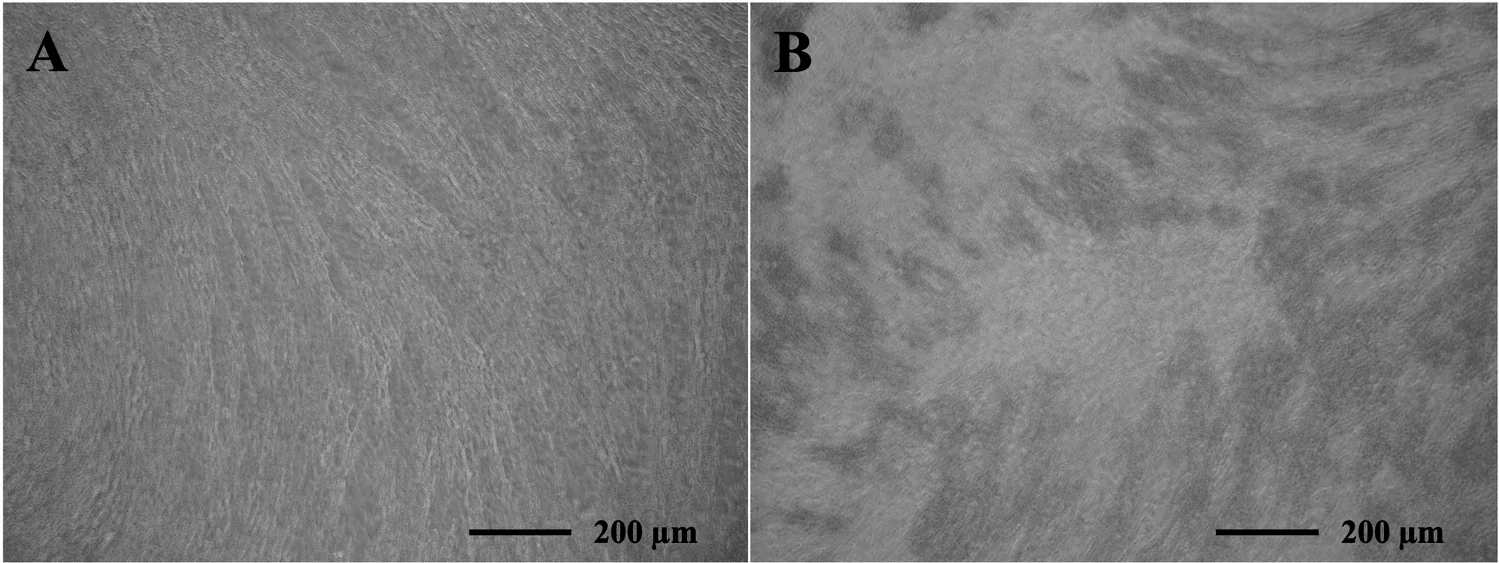
Microscopic images of MC3T3-E1 cells on day 28 supplemented with AA + DX + β-GP (*A*) and AA + β-GP (*B*). Dark stripes could be seen in the cell layers of both aforementioned groups.

Three days after osteogenic differentiation was induced, the first morphological changes could be observed using light microscopy. The cells supplemented with AA began to exhibit a more elongated, fibroblastic form (Fig. 1*B*, *C*, *E*, and *F*). In contrast, the cells incubated in cell culture medium without AA supplementation appeared cuboidal and resembled a cobblestone road pattern (Fig. 1*A* and *D*). These changes intensified until day 6 and then stagnated until day 28. On day 14, cells supplemented with AA + β-GP and AA + DX + β-GP began showing darker stripes that were visible for the next 14 d, until the end of the experiment (Fig. 2). After fixation on days 14, 21, and 28, the cell layer from the two aforementioned groups started to appear cloudy and milky.

### Qualitative results of Alizarin Red and von Kossa Staining

**Figure. 3:**
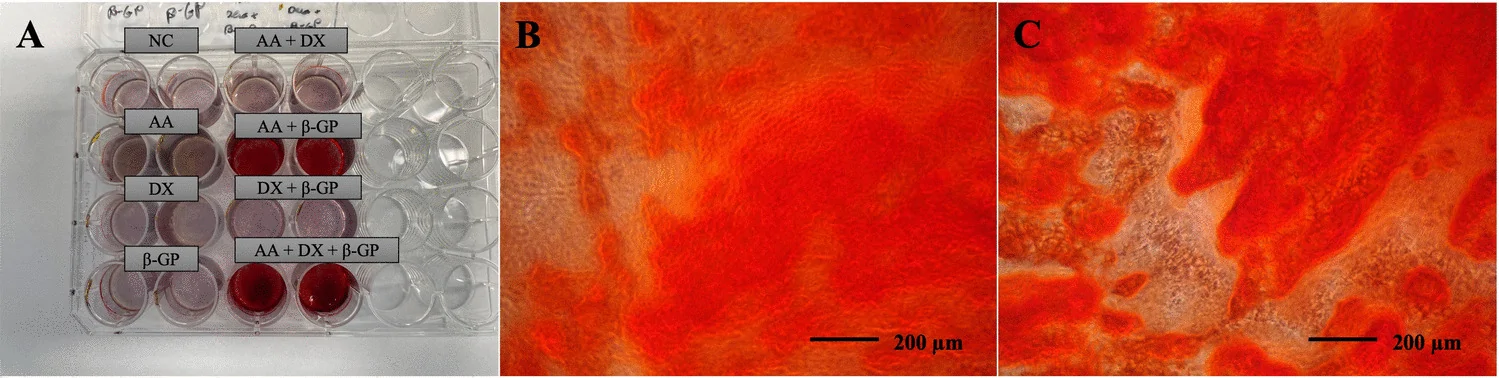
AR staining of the cell layers from MC3T3-E1 cells on day 28. Macroscopic image of the 24-well plate with MC3T3-E1 cells with positive staining signal for the groups AA + β-GP and AA + DX + β-GP (*A*). Microscopic image on day 28 of the cell layer for the AA + DX + β-GP group (*B*). Microscopic image on day 28 of the cell layer for the AA + β-GP group (*C*). *NC*, negative control; *AA*, ascorbic acid; *DX*, dexamethasone; *β-GP*, beta-glycerophosphate.

On day 7, no group showed a positive AR signal. By day 14, two groups, AA + β-GP and AA + DX + β-GP, showed positive AR staining. AA + DX + β-GP showed slightly stronger staining. The staining intensity of these two groups increased further on days 21 and 28, with the AA + DX + β-GP group always showing greater staining intensity. The other groups did not show a positive staining signal throughout the experiment. Consistent results in terms of staining intensity at the same time points for the same groups were obtained with VK staining (Fig. 3).

The stripes described previously started to appear on day 14 in groups AA + β-GP and AA + DX + β-GP. They became more apparent by day 28 and showed strong staining intensity with both stains, AR and VK. This indicates parts of the cell layer with a high degree of mineralization (Figs. 3*B* + *C* and 4*B* + *C*).

**Figure. 4:**
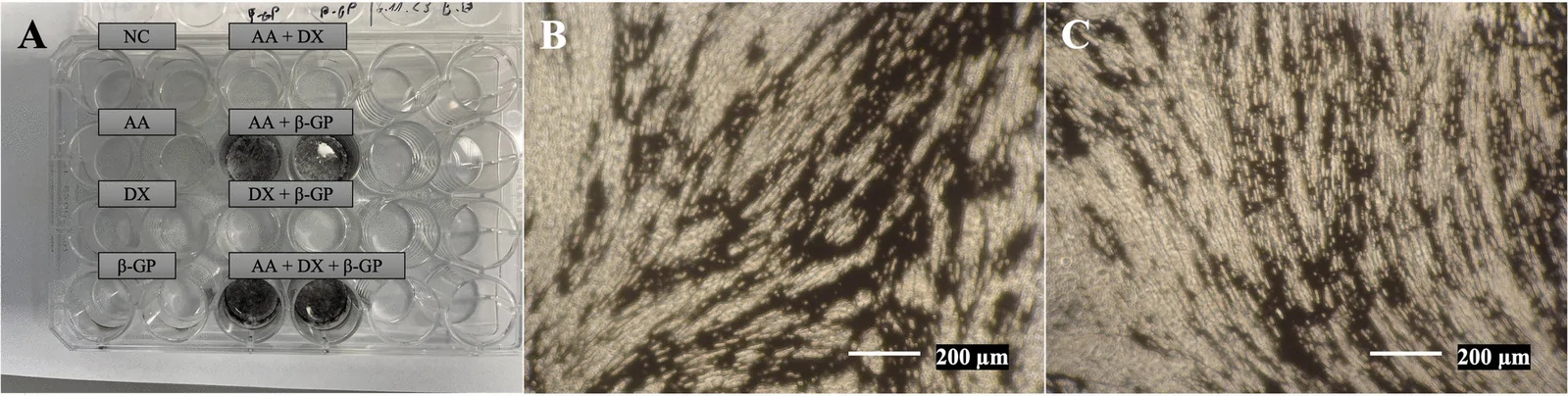
VK staining of the cell layers from MC3T3-E1 cells on day 28. Macroscopic image of the 24-well plate with MC3T3-E1 cells with positive staining signal for the groups AA + β-GP and AA + DX + β-GP (*A*). Microscopic image on day 28 of the cell layer for the AA + DX + β-GP group (*B*). Microscopic image on day 28 of the cell layer for the AA + β-GP group (*C*). *NC*, negative control; *AA*, ascorbic acid;* DX*, dexamethasone; *β-GP*, beta-glycerophosphate.

### Quantification of the Alizarin Red staining

**Figure. 5:**
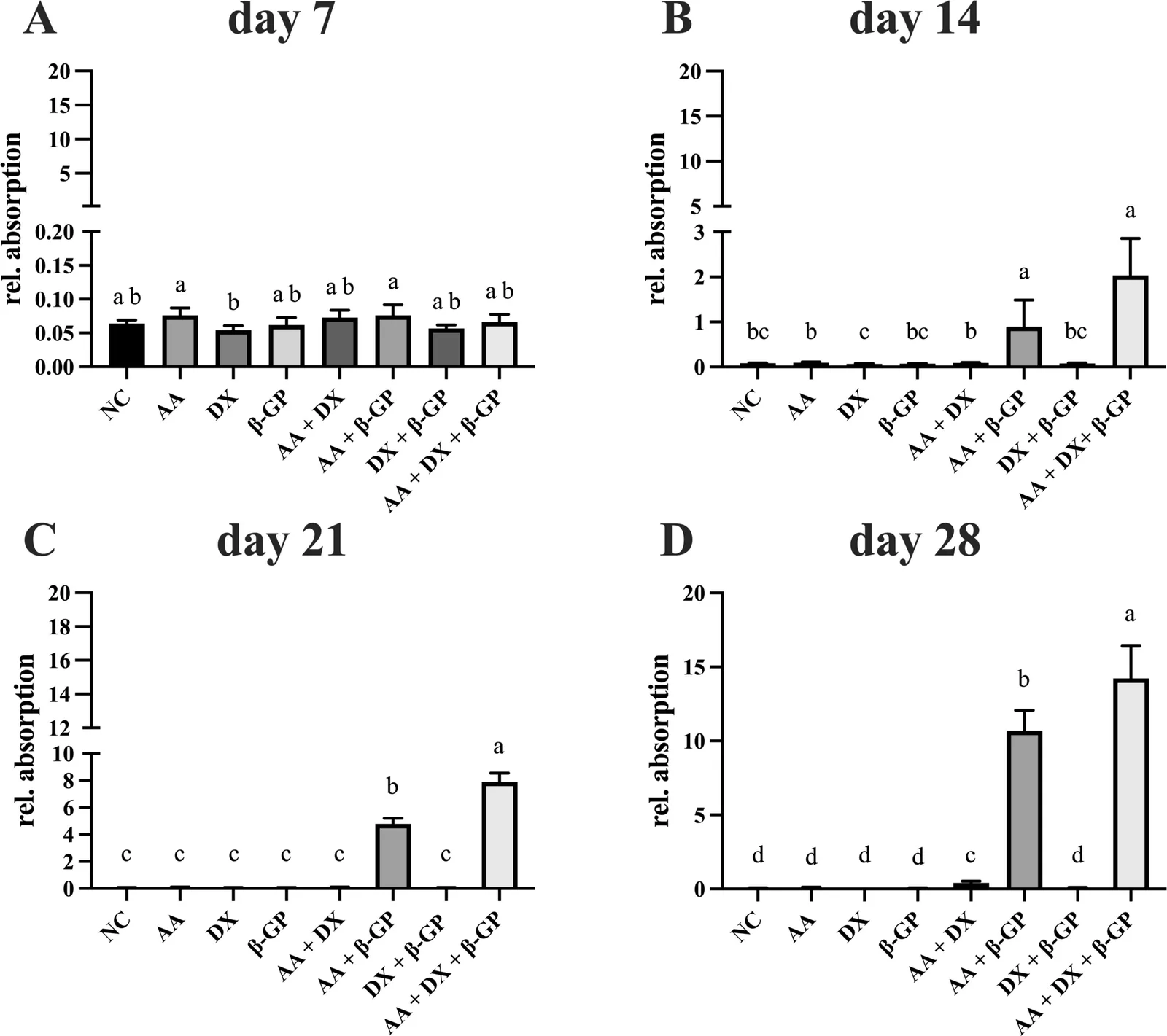
Photometric quantification (405 nm) of the eluted AR-calcium complex on days 7 (*A*), 14 (*B*), 21 (*C*), and 28 (*D*). *NC*, negative control; *AA*, ascorbic acid; *DX*, dexamethasone; *β-GP*, beta-glycerophosphate. Significant statistical differences (*p* < 0.05) between groups are illustrated with compact letter display from GraphPad Prism Version 10.2.3. *n* = 5.

Unlike VK, the AR stain can be eluted from the cell layers under acidic conditions and quantified photometrically. On day 7, significant differences in staining were observed between the AA and AA + β-GP groups compared to the DX group. These differences appeared without a macroscopically visible positive stain in any of the groups (Fig. 5*A*). On day 14, the staining intensity was higher in the AA + β-GP and AA + DX + β-GP groups compared to all other groups (*p* < 0.05). DX exhibited significantly lower relative absorption compared to groups supplemented with AA and AA + DX (Fig. 5*B*). On day 21, the AA + β-GP and AA + DX + β-GP groups showed significant differences from each other and from all other groups (Fig. 5*C*). The same significant differences were observed on day 28 between the two aforementioned groups. One difference compared to day 21 was that the AA + DX group showed a slightly positive red stain in the macroscopic assessment. This stain was also significantly higher in the quantification of relative absorption compared to the NC, AA, DX, β-GP, and DX + β-GP groups (Fig. 5*D*). In summary, the supplement cocktail containing all three supplements exhibited the highest relative absorption throughout the entire experiment. The difference in absorption between the AA + β-GP and AA + DX + β-GP groups remained stable from day 14 onward.

### Scanning electron microscopy

**Figure. 6:**
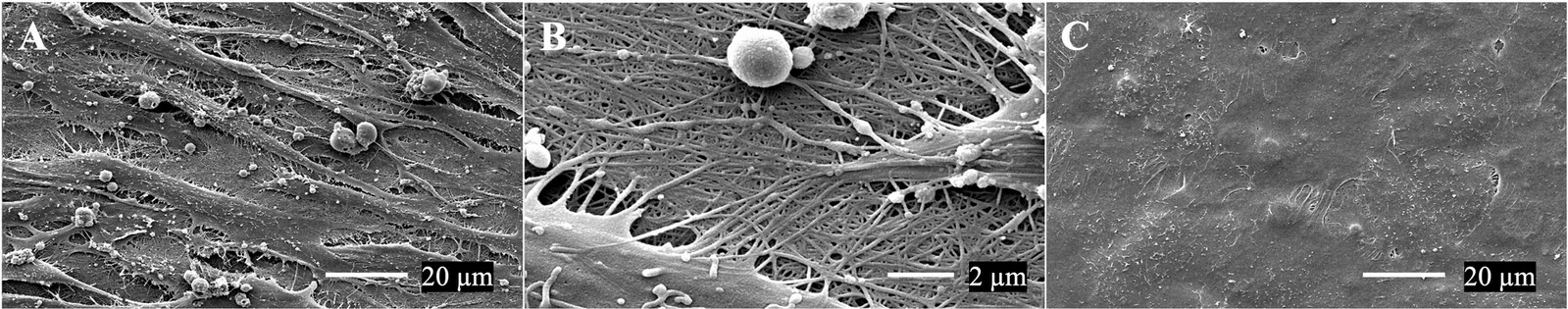
SEM images of different supplement groups on day 7. MC3T3-E1 cell morphology on day 7 with AA + DX + β-GP supplementation (*A* + *B*), MC3T3-E1 cell morphology on day 7 with DX + β-GP supplementation (*C*).

**Figure. 7:**
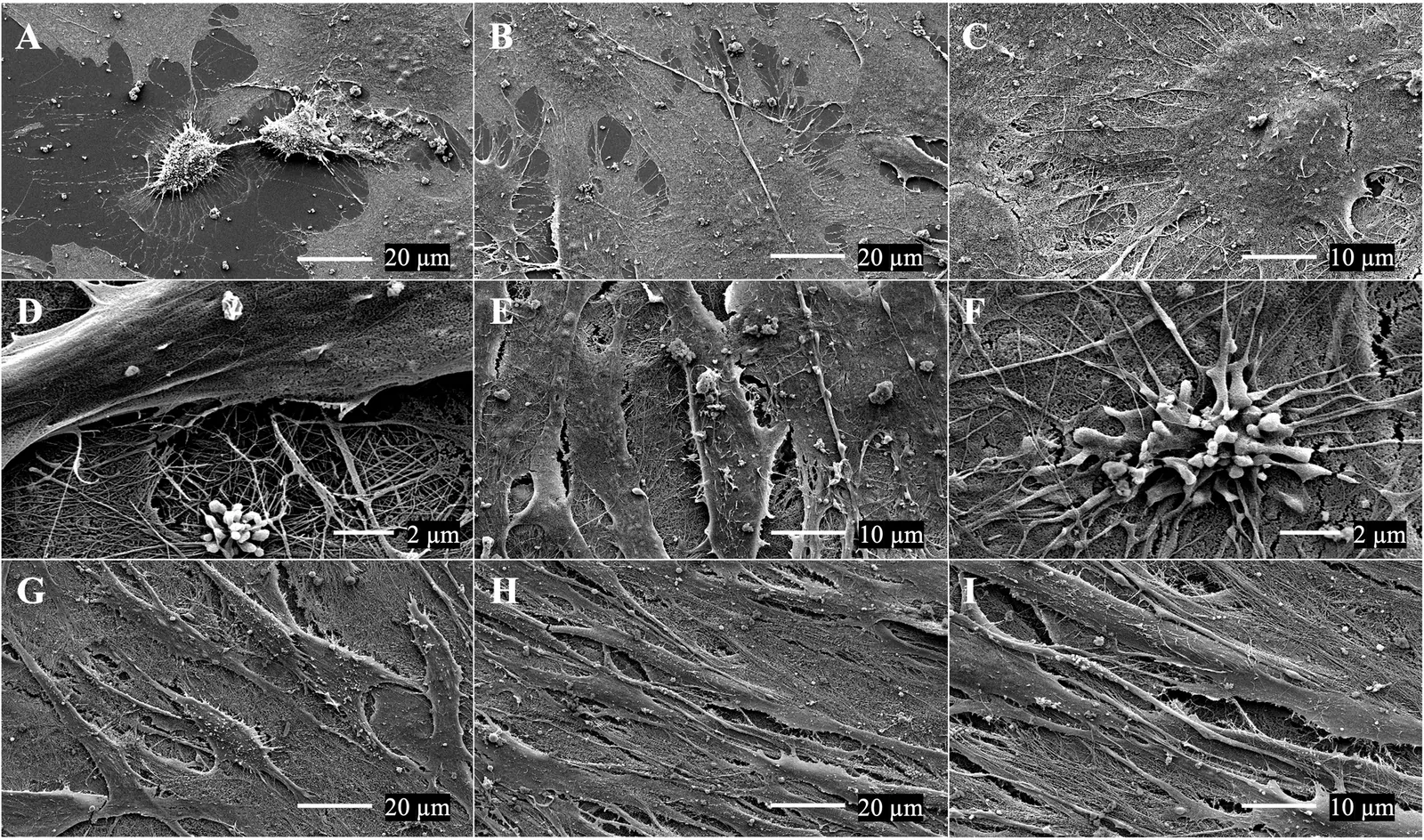
SEM images of the fiber development of MC3T3-E1 cells supplemented with AA alone on days 0–6. Before starting the differentiation process on day 0 (*A*), day 1 (*B*), beginning development of fibers on day 2 (*C*), day 3 (*D*), day 4 (*E*), possible excretion of the fibers (*F*), day 5 (*G*), and day 6 (*H* + *I*).

To gain a more detailed understanding of the cells’ morphology, a scanning electron microscopy (SEM) analysis was performed on days 7, 14, 21, and 28. On day 7, groups supplemented with AA alone or in any combination exhibited an abundance of fibers in the intercellular spaces (Fig. 6*A* + *B*). These fibers had a diameter of approximately 100 nm. No fibers were visible in the AA-free groups (Fig. 6*C*). Another observation was that cells tended to grow in layers under AA supplementation. The cells exhibited comparable morphology to that observed in the light microscopy analysis. Cells supplemented with AA grew in a fibroblastic/longitudinal shape, whereas cells supplemented without AA grew in a cuboidal shape (Fig. 6). In the absence of AA, a dense cell layer with minimal intercellular spaces could be seen (Fig. 6*C*). The morphology of the fiber layer remained stable until day 28. To better understand the early development of the fibers, we analyzed the NC and AA groups daily from days 0 to 6 (Fig. 7). To exclude the possibility that the observed fibers were a result of the collagen coating performed prior to seeding the cells, we first compared a native cover slip with a collagen-coated cover slip, both of which were free of cells. Both cover slips looked similar, and no fibers were visible. Therefore, the fibers identified during the SEM analysis did not originate from the collagen coating. The first appearance of fibers could be observed on day 2 in the AA group (Fig. 7*C*). As before, the NC group did not show any fibers. Over the next 5 d, the cells supplemented with AA began stacking in multiple layers, and the amount of fibers increased in the intercellular spaces (Fig. 7*D*–*I*).

### Energy-dispersive X-ray analysis

Because of the negative results regarding calcium and phosphate deposition in AR and VK staining, no EDX analysis was performed on day 7. On day 14, no calcium was detectable in any group. Small amounts of phosphorus were found in each group. The amounts of phosphorus measured on days 14 and 21 (approx. 0.5–0.8 wt-%) were slightly above the EDX analysis detection limit of about 0.5 wt-%. On day 21, calcium could only be detected in one out of five analyzed areas of the NC. Again, no calcium was detected in any other group.

**Figure. 8:**
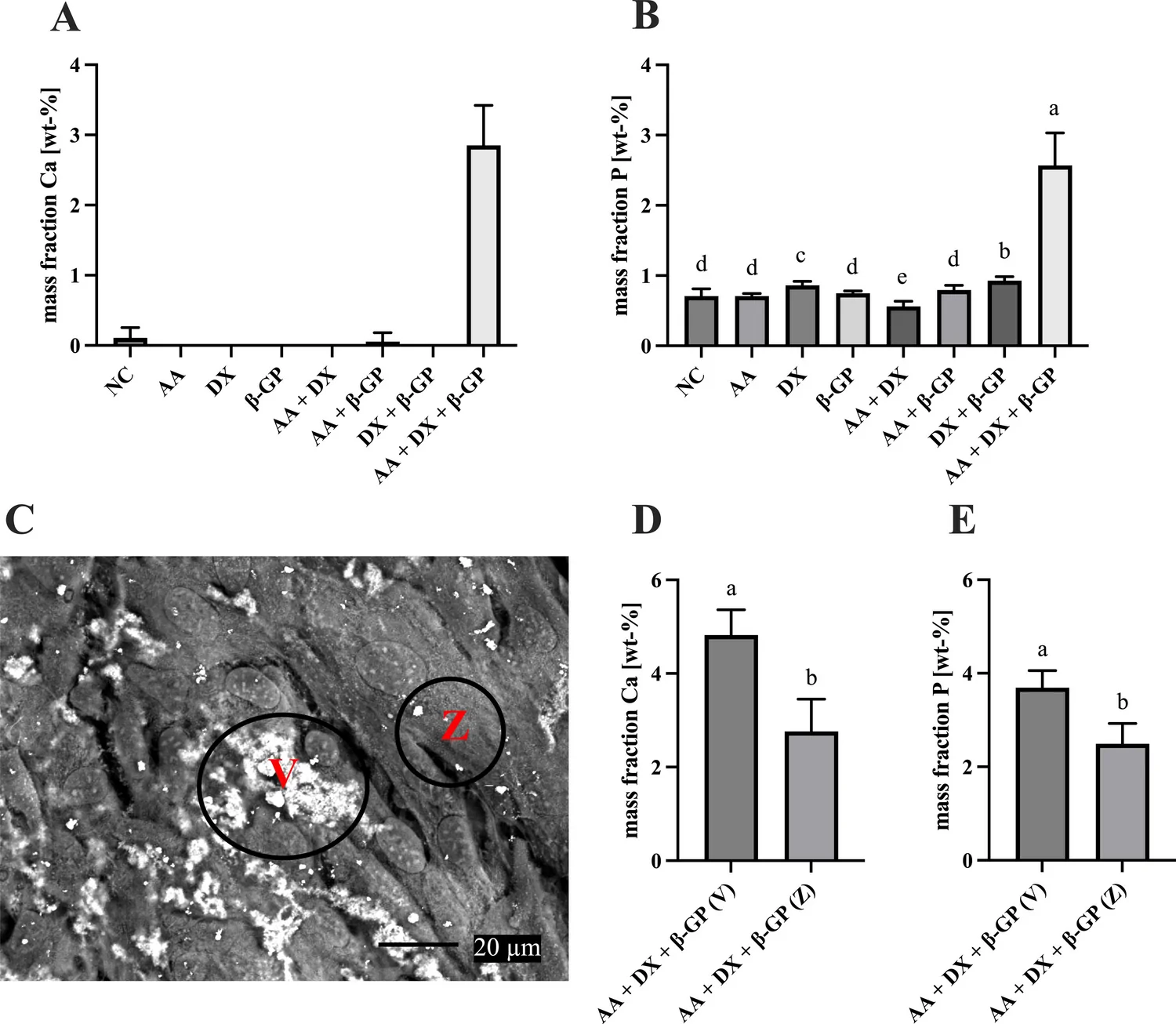
EDX analysis on day 28 for all groups (*A *+ *B*) and comparison of regions containing vesicle-like structures (*V* in *C*) and areas without vesicle-like structures (*Z* in *C*) in the BSE mode of the SEM (*C*) in the group supplemented with AA + DX + β-GP on day 28 (D + E). *NC*, negative control; *AA*, ascorbic acid; *DX*, dexamethasone, *β-GP*, beta-glycerophosphate. Significant statistical differences (*p* < 0.05) between groups are illustrated with compact letter display from GraphPad Prism Version 10.2.3.

On day 28, a significant difference in phosphorus content was observed between the AA + DX + β-GP group (2.57 ± 0.47 wt-%) and all other groups. Groups supplemented with DX + β-GP (0.93 wt-% ± 0.06) and DX alone (0.83 wt-% ± 0.06) differed from any other group. The group supplemented with AA + DX had the lowest amount of phosphorus (0.56 wt-% ± 0.07), which was significantly less than any other group (Fig. 8*B*). The highest total amount of calcium, 2.85 wt-% ± 0.57, was detected in the AA + DX + β-GP group. No significant differences in calcium were observed between any of the groups. Calcium was not detected in the AA, DX, β-GP, AA + DX, or DX + β-GP group (Fig. 8*A*). Interestingly, we observed vesicle-like structures on the cell membrane in the backscattered electron (BSE) mode of the SEM (Fig. 8*C*). We analyzed five areas containing vesicle-like structures (Fig. 8*C*, marked with *V*) and five areas free of these structures (Fig. 8*C*, marked with *Z*) from the AA + DX + β-GP group. The scanned vesicles exhibited significantly higher concentrations of calcium and phosphorus than the areas without them. We measured 4.82 ± 0.54 wt-% vs. 2.76 ± 0.69 wt-% for calcium and 3.69 ± 0.37 wt-% vs. 2.49 ± 0.44 wt-% for phosphorus (Fig. 8*D* + *E*).

### Sirius Red staining

**Figure. 9:**
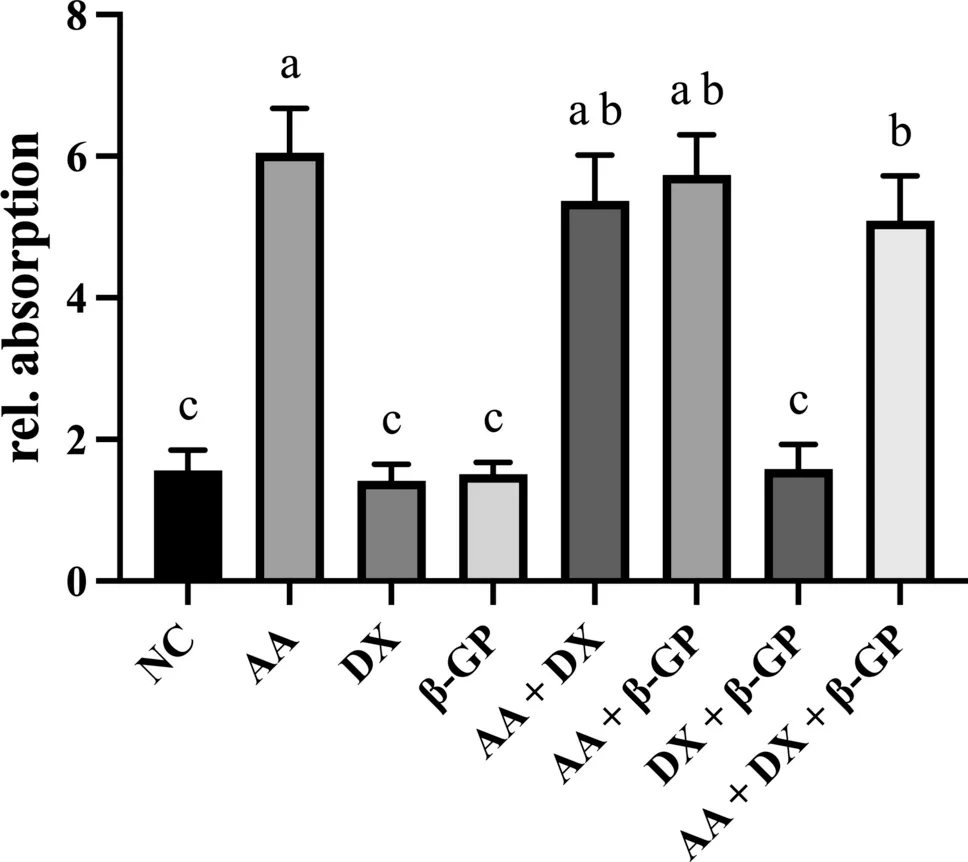
Photometric quantification (540 nm) of the eluted SR stain on day 7. The groups containing AA alone or in combination showed significant differences in the number of relative absorption compared to the groups without AA. The combination of all three supplements showed a slightly but significantly reduced secretion of collagen compared to the AA-supplemented group. *NC*, negative control; *AA*, ascorbic acid; *DX*, dexamethasone; *β-GP*, beta-glycerophosphate. Significant statistical differences (*p* < 0.05) between groups are illustrated with compact letter display from GraphPad Prism Version 10.2.3. *n* = 5.

Collagen secretion was visualized by SR staining on day 7. The staining revealed significantly higher levels of collagen in groups containing AA alone or in combination with other substances compared to AA-free groups (Fig. 9). Relative absorption in groups containing AA was about three times higher than in groups without AA. Among the groups containing AA, the group supplemented with AA alone showed higher relative absorption than the groups supplemented with all three supplements. No significant difference was detected between the groups supplemented with AA + DX or AA + β-GP compared to AA alone or the triple combination.

### Gene analysis - Comparison of the qPCR results between the negative control groups over time

The process of osteogenic differentiation involves complex genetic mechanisms. To determine how the supplements influence these mechanisms, we performed a qPCR analysis of typical osteogenic marker genes. First, we compared the NC of each day to the NC of day 7 to determine whether the MC3T3-E1 cells undergo spontaneous differentiation in standard medium without the addition of osteogenic factors. A significant upregulation of most osteogenic marker genes was observed over the course of the experiment (Table 3.). This indicates spontaneous upregulation of osteogenic genes over time, despite the absence of mineralization detected via AR and VK staining. To focus on the real effect of the supplements in the following qPCR timepoints, we only compared the supplemented groups with the NC from the same day.

**Table 3.** RQ values of qPCR analysis of the NC groups

	NC day 7 RQ mean	NC day 14 RQ mean (RQ range)	NC day 21 RQ mean (RQ range)	NC day 28 RQ mean (RQ range)
*ALPL*	1.00	**2.96 (2.56–3.38)**	**5.12 (2.99–7.52)**	**4.24 (2.34–6.49)**
*IbSp*	1.00	**2.99 (2.55–3.45)**	**5.10 (3.64–6.71)**	**5.69 (2.71–9.20)**
*SP7*	1.00	1.91 (1.28–2.61)	1.63 (1.03–2.32)	1.99 (1.80–2.20)
*RUNX2*	1.00	**1.60 (1.43–1.77)**	**2.79 (2.12–3.51)**	**2.75 (2.34–3.18)**
*BGLAP*	1.00	**3.85 (3.16–4.57)**	**15.97 (4.74–29.49)**	**6.40 (4.44–8.55)**
*Col1a2*	1.00	1.75 (1.03–2.58)	**2.27 (2.00–2.56)**	**2.71 (2.17–3.29)**

The NC group on day 7 was compared with the NC groups on days 14, 21, and 28. Significant statistical differences were calculated based on the ΔCT values and are written in bold

### Comparison of the qPCR results on day 7

By day 7, all genes in the group that received all three supplements were significantly upregulated (Table 4). The other groups containing AA alone or in combination with DX or β-GP showed significantly upregulated genes, except for *Col1a2*. The two genes *BGLAP* and *IbSp* showed tremendous upregulation, with RQ values ranging from 100 to 144 for *BGLAP* and from 30 to 60 for *IbSp*. No upregulation of any gene of interest could be detected in the β-GP group. In the DX group, only *IbSp* was upregulated, with an RQ value of 4.26 (2.47–6.31). The group containing DX and β-GP in the culture medium showed upregulation of *SP7* and *IbSp* expression. In summary, on day 7, only cells supplemented with AA showed induction of most, if not all, osteogenic marker gene expression. Cells supplemented with DX or β-GP alone or in combination failed to upregulate most genes, particularly important ones in the early phase of osteogenic differentiation: *RUNX2* and *SP7*.

**Table 4.** RQ values of qPCR analysis of all groups on day 7

	NC day 7 RQ mean	AA day 7 RQ mean (RQ range)	DX day 7 RQ mean (RQ range)	β-GP day 7 RQ mean (RQ range)
*ALPL*	1.00	**6.69 (4.47–9.16)**	1.25 (0.90–1.64)	1.36 (1.02–1.74)
*IbSp*	1.00	**29.62 (26.35–33.02)**	**4.26 (2.47–6.31)**	1.32 (0.94–1.75)
*SP7*	1.00	**5.81 (3.91–7.99)**	1.91 (1.28–2.62)	1.85 (1.11–2.72)
*RUNX2*	1.00	**3.14 (2.54–3.79)**	1.25 (0.91–1.62)	1.24 (0.82–1.72)
*BGLAP*	1.00	**124.60 (74.09–181.84)**	0.68 (0.43–0.96)	0.92 (0.70–1.17)
*Col1a2*	1.00	5.74 (4.53–7.03)	0.89 (0.76–1.01)	0.99 (0.82–1.17)
	AA + DX day 7 RQ mean (RQ range)	AA + β-GP day 7 RQ mean (RQ range)	DX + β-GP day 7 RQ mean (RQ range)	AA + DX + β-GP day 7 RQ mean (RQ range)
*ALPL*	**4.96 (2.67–7.74)**	**5.31 (2.90–8.21)**	1.66 (1.27–2.08)	**5.82 (2.94–9.15)**
*IbSp*	**47.95 (19.82–85.39)**	**31.39 (15.70–51.26)**	**6.05 (5.54–6.57)**	**61.66 (40.68–85.27)**
*SP7*	**5.09 (3.08–7.44)**	**8.53 (6.74–10.45)**	**2.23 (1.57–2.98)**	**7.89 (5.97–10.00)**
*RUNX2*	**2.85 (2.06–3.72)**	**4.30 (2.25–6.84)**	1.84 (1.71–1.98)	**3.11 (1.46–5.08)**
*BGLAP*	**118.24 (65.28–180.89)**	**100.04 (59.40–147.19)**	1.01 (0.33–1.84)	**144.19 (67.03–234.33)**
*Col1a2*	4.52 (2.15–7.29)	6.13 (2.27–10.83)	1.84 (1.13–2.67)	**7.76 (5.66–10.07)**

The NC group was compared with all other groups on day 7. Significant statistical differences were calculated based on the ΔCT values and are written in bold

### Comparison of the qPCR results on day 14

On day 14, the groups supplemented with AA alone or in combination again showed upregulation of most marker genes. In the group supplemented with AA + DX + β-GP, all genes were significantly upregulated, except for the early marker gene RUNX2, which showed no significant upregulation (Table 5). Similar results were observed for the AA and AA + β-GP groups (Table 5). The group supplemented with AA + DX showed upregulation of all six genes (Table 5). In the other groups containing DX or β-GP alone, only *IbSp* (DX) and *ALPL* (β-GP) showed slight upregulation. The combination of these two supplements increased the expression of *ALPL* (RQ 1.88 (0.90–3.14)), *IbSp* (RQ 4.44 (3.47–5.48)), and *BGLAP* (RQ 4.27 (1.98–7.25)) (Table 5). In summary, as on day 7, cells supplemented with AA alone or in combination showed upregulation of most, if not all, marker genes. The other cells, which were not supplemented with AA, again failed to increase expression in most of the tested genes.

**Table 5.** RQ values of qPCR analysis on day 14

	NC day 14 RQ mean	AA day 14 RQ mean (RQ range)	DX day 14 RQ mean (RQ range)	β-GP day 14 RQ mean (RQ range)
*ALPL*	1.00	**2.27 (1.68–2.91)**	1.24 (1.13–1.35)	**1.70 (1.51–1.90)**
*IbSp*	1.00	**6.79 (4.44–9.49)**	**4.13 (3.56–4.73)**	1.51 (0.64–2.56)
*SP7*	1.00	**4.04 (3.09–5.06)**	1.24 (0.80–1.74)	1.15 (0.82–1.50)
*RUNX2*	1.00	1.73 (1.00–2.59)	1.30 (0.74–1.94)	0.89 (0.67–1.13)
*BGLAP*	1.00	**44.51 (39.20–50.04)**	1.19 (1.04–1.35)	1.11 (0.95–1.27)
*Col1a2*	1.00	**3.07 (2.47–3.72)**	0.98 (0.79–1.18)	0.83 (0.48–1.25)
	AA + DX day 14 RQ mean (RQ range)	AA + β-GP day 14 RQ mean (RQ range)	DX + β-GP day 14 RQ mean (RQ range)	AA + DX + β-GP day 14 RQ mean (RQ range)
*ALPL*	**3.16 (2.65–3.69)**	**2.64 (1.88–3.49)**	**1.88 (0.90–3.14)**	**1.94 (1.50–2.43)**
*IbSp*	**14.69 (12.18–17.36)**	**7.56 (4.84–10.73)**	**4.44 (3.47–5.48)**	**15.40 (11.77–19.34)**
*SP7*	**3.52 (1.93–5.45)**	**4.11 (3.16–5.14)**	1.66 (1.22–2.14)	**4.10 (3.23–5.03)**
*RUNX2*	**3.56 (1.32–6.58)**	2.63 (1.73–3.65)	1.50 (1.10–1.93)	2.69 (1.84–3.64)
*BGLAP*	**84.84 (36.48–142.10)**	**48.41 (33.95–64.27)**	**4.27 (1.98–7.25)**	**85.56 (28.53–154.61)**
*Col1a2*	**4.04 (2.80–5.40)**	**2.99 (2.38–3.65)**	1.54 (0.84–2.37)	**3.54 (2.47–4.72)**

The NC group was compared with the other groups. Significant statistical differences were calculated based on the ΔCT values and are written in bold

### Comparison of the qPCR results on day 21

Groups supplemented with AA + β-GP and AA + DX + β-GP exhibited increased expression of the *IbSp*, *SP7*, and *BGLAP* genes on day 21. Compared to day 14, the expression of *ALPL*, *RUNX2*, and *Col1a2* was downregulated in both groups (Tables 5 and 6). The AA monosupplemented group and the AA + DX group had similar results, except that Col1a2 was still significantly upregulated (Table 6). Only *IbSp* (RQ 4.26 (3.90–4.64)) showed significant upregulation in the DX monosupplemented group. The β-GP-supplemented group did not show increased expression of any gene. The DX + β-GP group only showed upregulation of the *IbSp* (RQ 4.13 (3.04–5.31)) and *SP7* (RQ 2.28 (1.77–2.83)) genes. Cells that showed positive AR and VK staining on day 21 only exhibited three upregulated genes (*IbSp*, *SP7*, and *BGLAP*) at this time point (see “Qualitative results of Alizarin red and von Kossa staining” and Table 6). RQ values for some genes tended to be lower on day 21 than on days 7 and 14 (Tables 4, 5, 6). For example, *IbSp* in cells supplemented with AA + DX + β-GP showed RQ values of 8.12 (6.49–9.86) on day 21 and 61.66 (40.68–85.27) on day 7 (Tables 4 and 6).

**Table 6.** RQ values of qPCR analysis of all groups on day 21

	NC day 21 RQ mean	AA day 21 RQ mean (RQ range)	DX day 21 RQ mean (RQ range)	β-GP day 21 RQ mean (RQ range)
*ALPL*	1.00	1.44 (1.23–1.66)	0.88 (0.50–1.30)	0.95 (0.71–1.21)
*IbSp*	1.00	**6.55 (4.52–8.86)**	**4.26 (3.90–4.64)**	1.29 (1.10–1.49)
*SP7*	1.00	**5.28 (3.11–7.75)**	1.72 (1.02–2.52)	1.24 (1.17–1.32)
*RUNX2*	1.00	1.24 (1.13–1.36)	0.75 (0.65–0.85)	0.71 (0.63–0.78)
*BGLAP*	1.00	**46.22 (18.23–79.34)**	1.71 (0.22–4.25)	1.88 (0.22–4.46)
*Col1a2*	1.00	**2.76 (1.95–3.67)**	1.08 (0.72–1.50)	1.02 (0.79–1.28)
	AA + DX day 21 RQ mean (RQ range)	AA + β-GP day 21 RQ mean (RQ range)	DX + β-GP day 21 RQ mean (RQ range)	AA + DX + β-GP day 21 RQ mean (RQ range)
*ALPL*	1.28 (0.99–1.60)	1.29 (0.99–1.63)	0.76 (0.59–0.95)	0.75 (0.53–0.98)
*IbSp*	**9.89 (6.08–14.23)**	**4.97 (4.50–5.46)**	**4.13 (3.04–5.31)**	**8.12 (6.49–9.86)**
*SP7*	**3.62 (3.43–3.82)**	**4.24 (2.02–6.84)**	**2.28 (1.77–2.83)**	**6.75 (2.60–11.96)**
*RUNX2*	1.61 (0.87–2.49)	2.19 (0.86–3.80)	1.62 (1.04–2.27)	1.05 (0.81–1.31)
*BGLAP*	**17.90 (6.59–32.68)**	**13.86 (5.55–24.91)**	0.88 (0.39–1.48)	**11.63 (5.19–20.04)**
*Col1a2*	**2.13 (1.31–3.09)**	1.48 (0.67–2.51)	1.06 (0.66–1.52)	1.52 (1.45–1.59)

The NC group on was compared with all other groups. Significant statistical differences were calculated based on the ΔCT values and are written in bold

### Comparison of the results qPCR on day 28

No marker genes showed significant upregulation in the β-GP group on day 28 (Table 5). In the DX-supplemented group, *IbSP* was the only gene that was significantly upregulated at all four timepoints of the experiment (Table 4, 5, 6, 7). The combination of the aforementioned supplements also only significantly upregulated *IbSP* (RQ 5.18 (2.79–8.09)). The groups supplemented with AA, AA + β-GP, and AA + DX + β-GP only showed two upregulated genes: *IbSP* and *BGLAP*. The group supplemented with AA + DX showed additional upregulation of the *SP7* gene. At this late time point, fewer genes seemed to be upregulated by the supplements. In the groups supplemented with AA + β-GP or AA + DX + β-GP, which were identified as mineralized via the previously described staining, only the late markers *IbSP* and *BGLAP* showed significant upregulation.

**Table 7.** RQ values of qPCR analysis of all groups on day 28

	NC day 28 RQ mean	AA day 28 RQ mean (RQ range)	DX day 28 RQ mean (RQ range)	β-GP day 28 RQ mean (RQ range)
*ALPL*	1.00	1.35 (1.19–1.52)	1.61 (1.20–2.06)	1.51 (1.32–1.71)
*IbSp*	1.00	**6.31 (4.82–7.92)**	**4.50 (2.81–6.39)**	1.40 (1.14–1.67)
*SP7*	1.00	3.07 (2.37–3.84)	1.71 (0.97–2.56)	1.11 (0.85–1.40)
*RUNX2*	1.00	1.24 (0.99–1.51)	1.04 (0.93–1.15)	1.03 (0.91–1.16)
*BGLAP*	1.00	**23.50 (16.66–31.09)**	1.82 (1.19–2.54)	1.39 (0.89–1.97)
*Col1a2*	1.00	1.92 (1.63–2.22)	2.10 (0.59–4.00)	2.30 (0.76–4.15)
	AA + DX day 28 RQ mean (RQ range)	AA + β-GP day 28 RQ mean (RQ range)	DX + β-GP day 28 RQ mean (RQ range)	AA + DX + β-GP day 28 RQ mean (RQ range)
*ALPL*	1.88 (1.54–2.24)	1.76 (1.07–2.53)	1.29 (0.77–1.88)	0.94 (0.61–1.31)
*IbSp*	**7.75 (4.50–11.66)**	**6.32 (2.68–10.63)**	**5.18 (2.79–8.09)**	**9.80 (8.23–11.45)**
*SP7*	**3.72 (2.01–5.69)**	3.57 (1.41–6.52)	2.31 (1.26–3.54)	3.25 (1.71–5.04)
*RUNX2*	1.24 (0.93–1.57)	1.23 (0.75–1.78)	0.96 (0.60–1.37)	1.07 (0.92–1.21)
*BGLAP*	**28.44 (16.04–42.80)**	**39.82 (23.24–59.73)**	1.81 (1.62–2.01)	**28.90 (17.63–42.02)**
*Col1a2*	2.72 (1.41–4.23)	3.30 (1.23–5.92)	1.78 (0.84–2.89)	1.47 (0.96–2.06)

The NC group was compared with all other groups. Significant statistical differences were calculated based on the ΔCT values and are written in bold

### Bone morphogenetic protein 2 gene expression

Throughout all four time points of the qPCR, no detectable expression of the *BMP2* gene was observed in any group.

### Trend of osteogenic marker genes over time

**Figure. 10:**
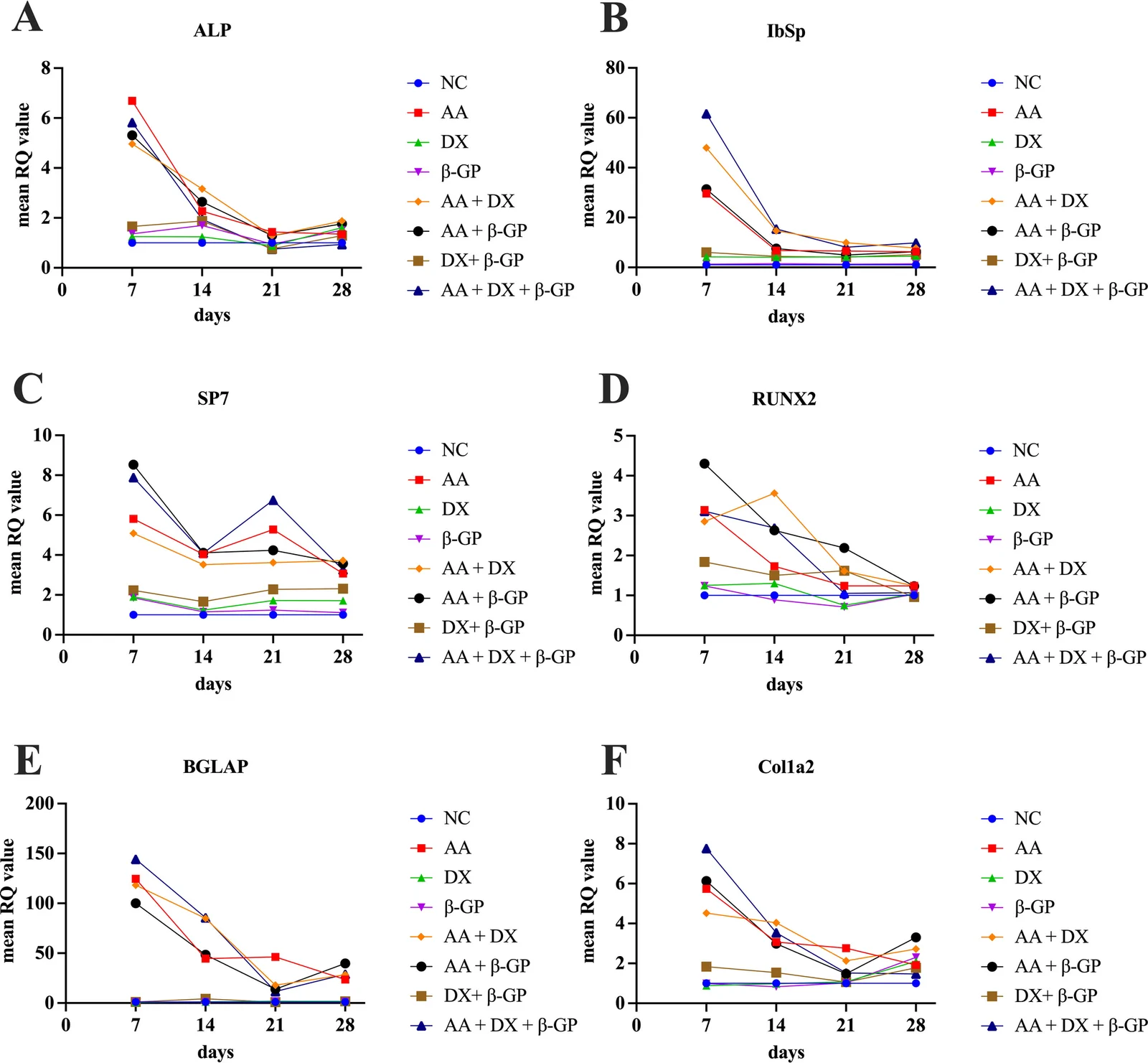
Mean RQ value (*n* = 3) trend of the osteogenic marker genes *ALPL* (*A*), *IbSp* (*B*), *SP7* (*C*), *RUNX2* (*D*), *BGLAP* (*E*), and *Col1a2* (*F*) over time. For reason of clarity, only the mean of the RQ value without the RQ range is displayed in this *graph*. Details can be found in Tables 4, 5, 6, and 7.

Examining the expression profile of each gene over time revealed that, in groups supplemented with DX and/or β-GP, almost all genes remained stable at all tested time points (Fig. 10). Compared to the other groups, these three groups formed a baseline that fluctuated around the RQ value of 1 from the NC (Fig. 10). All groups containing AA exhibited the highest RQ values on day 7 for all six genes (Fig. 10). After that, the RQ values declined until the end of the experiment. An exception was observed for *SP7*, which initially dropped on day 14 and then showed a second peak on day 21 (Fig. 10*C*). The highest fold changes were observed for the *IbSp* (30–60 RQ values) and *BGLAP* (100–144 RQ values) genes in groups supplemented with AA alone or in combination (Tables 4, 5, 6, 7). Over the following days, gene expression for these two genes decreased, though RQ values of 25–40 (*BGLAP*) and 6–10 (*IbSp*) were still observed on day 28 (Table 5). On day 7, the rest of the genes in these groups showed fold changes around 4–8, which decreased over time to RQ values within the range of the NC (Fig. 10).

## Discussion

In cellular bone research, the literature describes many different protocols for inducing the osteogenic differentiation of various cell types. Typically, osteogenic supplements are used for this purpose in various combinations and concentrations. One problem resulting from this heterogeneous approach is the lack of comparability between studies. Another issue is the lack of detailed studies on the direct influence of these supplements on cells. Often, the osteogenic supplements play a minor role in the study design. To address these issues, we examined the direct impact of three commonly used supplements (AA, DX, and β-GP) in various combinations on murine osteoprogenitor cells (MC3T3-E1) by evaluating morphology, mineralization, and osteogenic marker gene expression at four distinct time points.

When examining the influence of the supplements on cell morphology, we observed that the cells supplemented with AA had a fibroblastic shape under a light microscope. Cells in an AA-free culture medium exhibited a more cuboid shape. This finding aligns with a study by Park *et al*., who described the fibroblastic morphology of MC3T3-E1 cells after supplementation with the same three supplements at the same concentration (Park 2012). This initial observation of fibroblastic cell morphology was confirmed by SEM analysis. Furthermore, the cells supplemented with AA exhibited massive fiber production starting on day 2 in the intercellular spaces. This finding is consistent with those of other studies (Addison *et al*. 2015). To clearly identify the fibers, we performed SR staining, a sensitive and specific method for detecting and quantifying collagen (Tullberg-Reinert and Jundt 1999). Results of this staining revealed significantly higher collagen levels in all groups supplemented with AA than in groups supplemented with an AA-free culture medium. Additionally, we measured the caliber of the fibers using SEM, finding it to be around 100 nm. This measurement is comparable to those stated in the literature for collagen fibrils (Lüllmann-Rauch 2009). The ability of AA to act as a cofactor in hydroxylation during collagen production is well known (Heinrich *et al*. 2014). It plays an important role in the physiological process of osteogenic differentiation. Therefore, we conclude that supplementing with AA induces collagen production in cells (Franceschi and Iyer 1992). We were surprised by the large quantity of fibers produced in such a short period of time, as observed in the SEM analysis. It is possible that the fibroblastic morphology of the cells depends on the collagen fibers between the cells. These fibers may induce an environment or surface topography that favors the cells lining up and changing their shape (Wang *et al*. 2015; Wu *et al*. 2015).

When checking for mineralization of the cell layer, executing two stainings and looking for congruent, comparable results in both is an effective way to reduce the error rate associated with performing only one staining. In line with the literature, we used an additional method to analyze the mineralization, namely the EDX analysis (Bonewald *et al*. 2003). Our results showed positive, congruent, and comparable AR and VK staining results for the AA + β-GP and AA + DX + β-GP groups from days 14 to 28, indicating real mineralization. Throughout the experiment, the relative absorption value in the AR staining continuously rose for these groups, with the AA + DX + β-GP group having significantly higher values than the AA + β-GP group. These results align with those of other studies that showed positive AR staining for the AA + β-GP and AA + DX + β-GP groups (Hong *et al*. 2010; Luttrell *et al*. 2019). Another group reported positive VK staining results for human bone marrow cells on day 21 under β-GP monosupplementation at the same concentration used in this study. However, this could not be observed in our β-GP group’s VK staining results (Coelho and Fernandes 2000). The difference between these groups could be due to working with different cell types or slight differences in the staining protocols. A study by Czekanska *et al*. described the first positive AR staining on day 28 for MC3T3-E1 cells supplemented with AA (50 µg/ml), DX (10 nM), and β-GP (5 mM). Besides the β-GP concentration, this was the same setup used in this study (Czekanska *et al*. 2014). In light of our group’s recently published work, we decided to use a higher concentration of β-GP (Metzger *et al*. 2025). The 14-day time difference between the first positive AR staining in this study could be influenced by the cell culture media used or the differing concentrations of β-GP. In our study, we used α-MEM, whereas Czekanska *et al*. used DMEM. Orriss and colleagues emphasize that selecting the appropriate cell culture medium is essential for the outcome of an experiment. They demonstrated this in their experiment with primary mouse osteoblasts, which only formed significant bone formations when cultivated in α-MEM (Orriss *et al*. 2014). This observation is also in line with our own experience regarding the influence of different cell culture media on bone research (Brauer *et al*. 2016; Metzger *et al*. 2025). According to Orriss and co-workers, the higher concentrations of amino acids and nucleotides in α-MEM may be responsible for the media-specific influence on osteogenic differentiation (Orriss *et al*. 2014). In the context of staining methods like AR and VK, another important aspect is the probability of dystrophic mineralization due to high β-GP concentrations. Dystrophic mineralization has been reported when the β-GP concentration exceeds 5 mM (Orriss *et al*. 2014). Another group did not observe problems with dystrophic mineralization, even though the β-GP concentration was 10 mM (Koblenzer *et al*. 2022). Based on our experience, we did not observe dystrophic mineralization. However, to eliminate this potential issue, we recommend using 5 mM of β-GP in future studies.

We analyzed five areas of the cell layers from each group in the EDX analysis to detect calcium and phosphorus. Calcium was only detected in the group supplemented with all three supplements on day 28. This finding was surprising because we expected to detect calcium and phosphorus since day 14 in the groups supplemented with AA + DX + β-GP and AA + β-GP, consistent with the positive AR and VK staining results. To rule out any issues with our SEM device, we repeated the EDX analysis with a different SEM and obtained similar results. Problems that could alter the results of an EDX analysis include beam-induced damage to the probe, the tilting angle of the probe in relation to the beam, the roughness of the measured surface, and the organic phase of the bone (Vajda *et al*. 1998; Shirley and Jarochowska 2022). One possible explanation for these incongruent staining and EDX analysis results is that the electron beam could not penetrate deeply enough into the cell layer due to the multiple cell layers growing on top of each other. Another possibility is the inhomogeneous distribution of elements within the cell layer. This did not affect the staining method because the entire cell layer was stained. However, it made detection in the EDX analysis difficult and prone to error because only five random small areas in the probe were measured. Despite the challenges of conducting EDX analysis, a significant advantage of this method is the improved spatial resolution achieved in BSE mode of the SEM. This feature allows one to differentiate areas with a higher amount of calcium and phosphorus from areas where no mineralization occurred. This was demonstrated in this study for the AA + DX + β-GP group. These extracellular secretion areas with higher amounts of calcium and phosphorus, which we described as vesicle-like structures, were also observed in other studies and interpreted similarly as areas with high mineralization activity (Cao *et al*. 2016; Metzger *et al*. 2025).

Another important factor to consider when discussing the influence of supplements on osteogenic differentiation is the genetic processes that occur. Our results show that some osteogenic marker genes are spontaneously upregulated even when no osteogenic supplements are added. Although most osteogenic marker genes were significantly upregulated, no mineralization was detected in the NC at any time via any method (Kodama *et al*. 1981). This spontaneous differentiation at the gene level must be considered when interpreting the qPCR results from MC3T3-E1 cells. It shows that combining phenotypic and genotypic experiments is more advantageous than using just one or the other. Due to this spontaneous differentiation at the genetic level, an additional qPCR time point on day 0 should be performed to generate the baseline expression of the marker genes.

When examining the influence of the supplements alone or in combination, the following was observed: No upregulated osteogenic marker genes were observed in cells supplemented with β-GP alone over the entire experimental period. This indicates that β-GP alone cannot induce changes in the MC3T3-E1 cells’ genetic pattern. The main mechanism by which β-GP works in osteogenic differentiation is as a source of phosphorus for ALPL. One major event in osteoblast mineralization activity is the hydrolysis of phosphorus, so ALPL can be considered the key enzyme in bone formation (Garimella *et al*. 2006; Makris *et al*. 2023). Other research groups have proposed that β-GP has an additional influence on osteopontin expression (Beck & Knecht, 2003) and *BMP2* (Tada *et al*. 2011), but we did not observe any expression of *BMP2* in our study. Therefore, we conclude that MC3T3-E1 cells cannot induce the BMP2 gene when supplemented with β-GP or any of the other supplements under investigation. One reason for this discrepancy (Tada *et al*. 2011) could be the use of human dental pulp cells and Na_2_HPO_4_ and NaH_2_PO_4_ instead of MC3T3-E1 and β-GP. Another possible explanation is that the combination of AA and DX with Na₂HPO₄ and NaH₂PO₄ could have caused the upregulation of BMP2 expression, rather than the phosphate compounds alone (Tada *et al*. 2011).

For cells supplemented with DX alone, no significant changes in the genetic pattern besides *IbSp* were observable. This finding aligns with the results of other research groups on the influence of DX on osteoblastic cells (Ogata *et al*. 1995; Sodek *et al*. 1995). Many studies have attempted to elucidate the mechanism by which DX functions in the process of osteogenic differentiation; however, the exact mechanism has yet to be discovered. Due to the use of various cell types and experimental protocols with different DX concentrations and durations, conflicting data are reported in the literature (Langenbach & Handschel 2013). In this study, no clear osteogenic differentiation mechanism could be identified for DX monosupplementation. However, combining all three supplements produced better differentiation results than the AA + β-GP group. These results suggest that DX promotes osteogenic differentiation when combined with the other two supplements in our experimental setup. Combining DX and β-GP resulted in the upregulation of only *IbSp* throughout the entire experiment. Additionally, *ALPL*, *BGLAP*, and *SP7* exhibited significantly increased expression at various time points throughout the experiment. Although the combination of these two supplements increased the expression of some genes, *RUNX2* was not upregulated. This is astonishing because *RUNX2* is considered essential to the early phase of osteogenic differentiation (Nakashima *et al*. 2002; Eriksen 2010). Therefore, the upregulation of *ALPL*, *BGLAP*, and *SP7* appears to be mediated by pathways that are not related to *RUNX2*. As observed with *SP7* on day 21, this may occur via the MSX2 pathway, which is independent of *RUNX2* and is activated by *BMP2* (Matsubara *et al*. 2008). Since no *BMP2* expression was detected in our study, perhaps other BMP isoforms, rather than BMP2, are responsible for the observed upregulation of SP7 expression. Cells supplemented with AA alone or in combination produced very similar qPCR results at all time points. By day 7, every gene had been upregulated except *Col1a2* in the AA, AA + DX, and AA + β-GP groups. Although the *Col1a2* results showed no significant upregulation in these groups, a trend was visible in the qPCR results. Combined with the enhanced fiber production observed in the SEM analysis and the positive SR staining, it is likely that AA promotes collagen production. One seemingly illogical observation is the discrepancy between the large number of fibers detected in the SEM analysis and the non-significant increase in Col1a2 gene expression on day 7. This discrepancy could be explained by a halt in posttranscriptional RNA silencing when AA is introduced to the cells. Therefore, the mechanism of collagen production would not be driven by elevated gene expression, but rather by posttranscriptional processes. However, this hypothesis must be evaluated through additional experiments. Another possible explanation is that AA does not impact the Col1a2 gene expression levels but rather acts in the collagen fibrillogenesis process by reducing the iron molecule in the active center of lysyl- and prolyl hydroxylases, which are essential to collagen production, as described in the literature (Heinrich *et al*. 2014). Thus, it is possible that the Col1a2 gene expression level remains the same whether AA is present in the medium or not, and that elevated production is steered through the aforementioned process. Early osteogenic marker genes, such as *RUNX2*, and late marker genes, such as *IbSp* and *BGLAP*, were upregulated in cells supplemented with AA alone or in combination. These results suggest that an additional earlier time point, such as day 3, should be included in the qPCR analysis to observe the early phase of gene expression. By day 14, three out of four groups supplemented with AA exhibited increased expression of all genes except *RUNX2*. Based on this observation, the early phase of osteogenic differentiation has already ended on day 14 because the early osteogenic marker gene *RUNX2* is no longer upregulated. However, expression of other osteogenic marker genes, which are promoted by *RUNX2*, remained elevated (Eriksen 2010; Komori 2022). On days 21 and 28, the late marker genes *IbSp* and *BGLAP* are the only ones that remain upregulated in the mineralized groups AA + β-GP and AA + DX + β-GP, as well as in nonmineralized cells that were monosupplemented with AA on day 28 (Cao *et al*. 2016). As can be seen in the qPCR results, AA seems to play an essential role in the process of osteogenic differentiation. The influence of AA on osteogenic differentiation is described in the literature, but the results are heterogeneous. Some studies describe a direct influence of AA on the expression of genetic marker genes, such as *SP7*, while others describe an indirect influence through the promotion of collagen production, which then binds to integrin α1β2 (Takeuchi *et al*. 1996; Aghajanian *et al*. 2015). However, the gene expression profile of AA monosupplementation is almost identical to the expression profile of AA + β-GP and AA + DX + β-GP supplemented cells, yet mineralization only occurred when AA was combined with the other supplements from the aforementioned groups. This indicates that AA’s influence on inducing osteogenic differentiation is crucial yet insufficient for achieving morphological mineralization of the cells when supplemented alone.

## Conclusion

In conclusion, the combination of AA and β-GP is essential for inducing osteogenic differentiation in MC3T3-E1 cells. The combination of all three supplements produced the best results for inducing osteogenic differentiation over the 28-day monitoring period. The relative absorption in AR staining was higher compared to the AA + β-GP combination, and the EDX analysis revealed areas of mineralization in BSE mode of the SEM with significantly higher amounts of calcium and phosphorus compared to non-mineralized areas. Our experiments clearly demonstrate that AA plays a crucial role in collagen production, which is indispensable for osteogenic differentiation. Another important consideration when performing experiments on osteogenic differentiation is the timing of genetic and phenotypic changes in the cells. Genetic changes can be observed much earlier after differentiation induction, whereas phenotypic changes start to occur on day 14. These differences are important to keep in mind when planning and performing studies of this kind.

To clarify the exact influence of the supplements on processes during osteogenic differentiation in MC3T3-E1 cells, other experimental approaches should be employed to gain a deeper understanding of the supplements, such as transcriptomics. Understanding the exact influence of the supplements is crucial because they often form the basis of experimental designs in basic bone research and can drastically affect the results of experiments if not used properly.

## Data Availability

Data will be made available upon reasonable request to the corresponding author.

## Declarations

### Statement on clinical trial

Clinical trial number: not applicable.
